# Delineation of early attentional control difficulties in fragile X syndrome: Focus on neurocomputational changes

**DOI:** 10.1016/j.neuropsychologia.2006.12.005

**Published:** 2007

**Authors:** Gaia Scerif, Kim Cornish, John Wilding, Jon Driver, Annette Karmiloff-Smith

**Affiliations:** a Department of Experimental Psychology, University of Oxford, Oxford OX1 3UD, UK; b McGill University and Montreal Neurological Institute, Canada; c Royal Holloway, University of London, UK; d Institute of Cognitive Neuroscience, University College London, UK; e Developmental Neurocognition Lab, Birkbeck College, University of London, UK

**Keywords:** Atypical development, Attentional control, Neurocomputational changes

## Abstract

Fragile X syndrome (FXS) is due to the silencing of a single X-linked gene and it is associated with striking attentional difficulties. As FXS is well characterised at the cellular level, the condition provides a unique opportunity to investigate how a genetic dysfunction can impact on the development of neurocomputational properties relevant to attention. Thirteen young boys with FXS and 13 mental-age-matched typically developing controls performed a touch-screen-based search task that manipulated the similarity between targets and distractors and their heterogeneity in size. Search speed, path and errors were recorded as multiple measures of performance. Children did not differ in overall search speed or path when searching amongst distractors, but striking error patterns distinguished children with FXS from controls. Firstly, although clear markers of previously found targets remained on screen, children with FXS perseverated on touching previous hits more than typically developing controls, consistent with the well-documented inhibitory deficits in adults with the disorder. Secondly, they could accurately discriminate single target-distractor pairs, but, when searching a complex display, they touched distractors more often than control children when distractors were similar to targets and especially so when these were infrequent, highlighting difficulties in judging relative size and allocate attentional weight independently of stimulus frequency. Thirdly, their performance was also characterised by inaccuracies in pointing, suggesting additional motor control deficits. Taken together, the findings suggest that fragile X syndrome affects the early development of multiple processes contributing to efficient attentional selection, as would be predicted from an understanding of the neurocomputational changes associated with the disorder.

Fragile X syndrome (FXS) is the most common form of inherited mental retardation in males, with an incidence estimated at 1 in 4,000–9,000 (Crawford, Acuna, & Sherman, 2001). It is associated with the silencing of a single gene, the Fragile X Mental Retardation gene (FMR1, Verkerk et al., 1991). The cellular physiology and the cortical patterns of expression of the protein associated with FMR1 have been recently mapped out, making fragile X syndrome a unique model to investigate the relationships between the silencing of a single gene and the development of neurocognitive dysfunction (e.g., Jin & Warren, 2003; Reiss & Dant, 2003). Furthermore, an increasingly large number of individuals with the condition are now diagnosed early in childhood (Bailey, Roberts, Mirrett, & Hatton, 2001), highlighting the need to study processes leading to very early deficits in cognitive functioning.

Serious problems of inattention and hyperactivity are clinically diagnostic of fragile X syndrome across the lifespan (Cornish, Sudhalter, & Turk, 2004; Hagerman & Hagerman, 2002; Turk, 1998). Adults and older children with the syndrome differ from typically developing individuals and those with other genetic disorders in their inability to inhibit task-irrelevant repetitive responses (Cornish, Munir, & Cross, 2001; Munir, Cornish, & Wilding, 2000; Wilding, Cornish, & Munir, 2002). Recently, even toddlers with fragile X syndrome demonstrated difficulties in executive control (Scerif, Cornish, Wilding, Driver, & Karmiloff-Smith, 2004). When assessed with a touch-screen-based task that required searching for targets amongst a variable number of distractors that were more or less similar to the targets in size, toddlers with FXS repeatedly touched targets that they had already found, suggesting difficulties in inhibiting previously successful responses, a pattern that mirrored the errors by older children with FXS (Wilding et al., 2002). Deficits in inhibiting inappropriate eye-movements in an analogue of the antisaccade task were also demonstrated in infants with FXS as young as 12 months old (Scerif et al., 2005).

What are the neural correlates of these seeming life-long difficulties with executive control? Adult women with fragile X syndrome have shown dysfunctional activation of prefrontal and parietal cortices during multiple tasks requiring executive control (e.g., Cornish, Swainson, et al., 2004; Menon, Leroux, White, & Reiss, 2004; Tamm, Menon, Johnston, Hessl, & Reiss, 2002). In order to understand these findings at the systems level, it is crucial to appreciate the cellular pathophysiology of FXS at least in basic terms. The Fragile X Mental Retardation gene (FMR1) codes for a protein (FMRP) that plays a key role in the post-synaptic refinement of dendritic spine morphology following the excitation of metabotropic glutamatergic receptors, type I (Bagni & Greenough, 2005; Bear, Huber, & Warren, 2004). FMRP acts as a translational repressor by regulating translation of multiple dendritic mRNAs involved in synaptic development and function (Brown et al., 2001), so that loss of FMRP is associated with immature dendritic spine morphology (e.g., Irwin et al., 2002) and dysregulation of other neurotransmitter pathways (e.g., monoamines, Gruss & Braun, 2004; Zhang et al., 2005). These morphological and functional changes seem ubiquitous across cortex, but they may be particularly disruptive for the development of executive functions, because these are supported by circuits that rely more extensively on these structural changes and neuromodulatory functions, as seems to be the case for prefrontal cortices and the networks to which they belong (refer to Scerif et al., 2005; Scerif & Karmiloff-Smith, 2005, for further details on this argument). However, an understanding of the neurobiology of the syndrome also predicts that executive difficulties should not be the sole characteristic cognitive deficit in FXS: multiple cognitive processes may be affected by the changes in neurocomputational properties associated with FMR1 silencing and its related cascade of molecular events. Widespread effects of changes in low-level computational properties, rather than selective effects on specific high-level cognitive functions, have indeed already been suggested to account for the complex cognitive profile in fragile X syndrome (Cornish, Turk, et al., 2004). The challenge remains to understand why certain cognitive processes are more affected than others, and why deficits co-occur in this disorder.

Multiple deficits do indeed seem to accompany prominent executive difficulties in toddlers with FXS. Scerif et al. (2004) asked typically developing toddlers, toddlers with FXS and toddlers with Williams syndrome (WS, another genetic disorder characterised by attentional difficulties) to search for targets amongst distractors that varied in number and were either similar or dissimilar to targets in terms of size. Search performance by toddlers with FXS was characterised by striking repetitive errors on previously found targets compared to the other groups, but also by a larger number of erroneous touches on distractors compared to typically developing controls, with toddlers with WS producing the greatest number of such errors. This suggested atypical processing of target-distractor similarity for toddlers with FXS or WS during search, despite the fact that these children could accurately discriminate single target-distractor pairs. These errors were particularly surprising for toddlers with FXS, given that older children with FXS (aged between 8 and 15 years of age) never confused targets and distractors in a variant of this task that required target-distractor discriminations through a categorical distinction (vertically as opposed to horizontally oriented stimuli of two different colours) (Wilding et al., 2002), rather than requiring a relative size judgment (Scerif et al., 2004). However, the study design did not allow investigating in detail all possible sources of these difficulties for children with FXS, and we therefore aimed to do so here.

The relative salience of targets is affected both by whether distractors are similar to targets and by whether distractors can be grouped into homogeneous sets (Duncan & Humphreys, 1989; Humphreys, Quinlan, & Riddoch, 1989). In fact, the effects of these two manipulations of target salience can be relatively independent of each other. For example, some patients with visual agnosia are atypically affected by the similarity of targets and distractors, but not by distractor heterogeneity, presumably because disadvantages associated with the latter depend on different processes, such as the disruption of Gestalt grouping of distractors (Humphreys, Riddoch, Quinlan, Price, & Donnelly, 1992). Older children with FXS display relative strengths in perceptual grouping (Cornish, Munir, & Cross, 1999). Therefore, target-distractor similarity and distractor heterogeneity may vary in the extent to which they impact on the efficiency of search in fragile X syndrome. Employing heterogeneous search displays for the first time afforded an additional empirical question. Infrequent items in a search display appear relatively more salient because computations of salience depend on the difference between an element and any other elements in the display (Cave & Wolfe, 1990) and between that element and neighbouring elements (Wolfe, 1994). Differences in salience for infrequent items account for strong effects of relative ratios across distractor types, with infrequent distractors receiving greater attentional weight and appearing more salient than frequent ones (e.g., Shen, Reingold, & Pomplun, 2000). Therefore, employing search displays composed of heterogeneous distractors would enable us to test the degree to which children are affected by the relative salience of distractors regardless of their similarity to targets.

In sum, we sought to extend earlier characterisations of control difficulties in older children with FXS (Wilding et al., 2002) and in toddlers with FXS (Scerif et al., 2004) by investigating in detail search performance in young children with FXS, with a particular emphasis on all their errors and on assessing the effects of target and distractor salience. We therefore manipulated concurrently, for the first time, target-distractor similarity, distractor heterogeneity and the relative proportion of distractors of various types. Firstly, if young children with FXS display early difficulties analogous to those in older individuals with the syndrome, their search performance should be characterised by repetitive errors. Secondly, if their ability to evaluate target and distractor relative salience is also affected, they should be differentially more affected by target-distractor similarity and by the frequency of distractors.

## 1 Method

### 1.1 Participants

Families of children with fragile X syndrome were contacted through the Fragile X Society, the national family support group for people affected by fragile X syndrome in the UK, and they volunteered to take part in a larger longitudinal study on the development of attention in fragile X syndrome. Thirteen male children with FXS completed the present search task (age range = 41–60 months, mean chronological age = 53.1 months, *SD* = 5.9 months). Five of these children had never been tested with this experimental setup, while eight children had contributed to a different search study (reported by Scerif et al., 2004) 12 months prior to taking part to this new experiment. Assessing their developmental level with the BSIDM-II (Bayley, 1993) revealed a mean mental-age equivalent of 28.4 months (*SD* = 6.2 months, range = 23–39). The children were individually matched, by mental age equivalent within 1 month, to 13 typically developing children, all of whom had also been assessed with the BSIDM-II (MA controls henceforth, mean mental and chronological age = 29.1 months, *SD* = 5.1 months, mental and chronological age range: 24–38 months). There were therefore no significant differences in developmental level between the MA controls and the children with FXS. Control children were recruited from local nurseries and a database of parents in the London area. While five children had never been tested with the current experimental setup, eight control children had also contributed to the study reported by Scerif et al. (2004). Controls were recruited to this study on the basis of developmental level. This is because children with FXS performed well below their chronological age equivalent for the primary variables of interest in the current study, error types (Munir et al., 2000; Scerif et al., 2004; Wilding et al., 2002) and because we were specifically concerned with how patterns of performance by children with FXS deviated from what would be expected given their overall developmental delay.

### 1.2 Materials

**Fig. 1:**
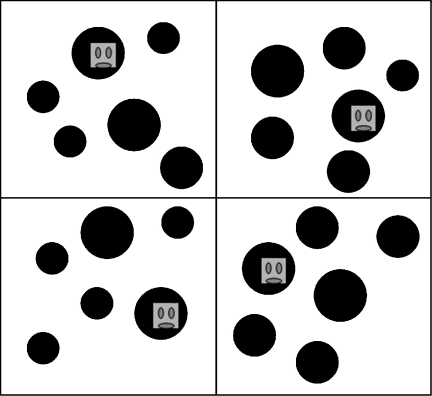
Sample test displays maintaining the target-distractor ratio in the original test displays. Top-left: heterogeneous distractors (mainly dissimilar), top-right: heterogeneous distractors (mainly similar), bottom-left: homogeneous distractors (dissimilar), bottom-right: homogeneous distractors (similar). Please note that display size was kept constant here, while the relative distractor sizes and their ratio varied (cf. Scerif et al., 2004).

During the demonstration phase, as well as practice and test runs, participants viewed stimuli on a 15 in. portable touch-screen connected to a portable laptop computer. Visual Basic was used to program presentation parameters. Large black target circles were randomly placed on an 8 × 4 light green grid. Viewed from a 30-cm distance, each target subtended 5.7° angle. Distractors were also black circles, subtending either 2.8° (small distractors, very dissimilar from the target) or 4.2° (medium distractors, more similar to the target). All search displays contained 10 large target circles and either no distractors (baseline condition) or 24 distractors (experimental runs). In two runs, distractors were heterogeneous. One display contained 18 small and 6 medium distractors (henceforth labeled “heterogeneous, infrequent similar”), whereas the other contained 18 medium and 6 small distractors (“heterogeneous, infrequent dissimilar”). In two runs (homogeneous distractor displays), these were 24 small (dissimilar) or medium (similar) circles, together with the large targets. These conditions are represented in Fig. 1.

### 1.3 Procedure

Procedures for recruitment and assessment of the patient and control group were approved by the Research Ethics Committee of Great Ormond Street Hospital and the Institute of Child Health, and were therefore performed in accordance with the 1964 Declaration of Helsinki. Caregivers gave their informed consent prior to inclusion in the study and commencement of the experimental procedure. Children were tested in a quiet room either at their nursery, home or in the Neurocognitive Development Unit Infant Testing Lab. They sat at a small table approximately 30 cm from the touch-screen, either on their caregiver's lap or on an appropriately sized chair. During four pre-test trials, toddlers were asked to “touch the big circle” on laminated cards displaying a single target and a distractor. They had to succeed in pointing to the large circle on the second acuity card for each distractor type to continue, ensuring that all could discriminate the two different types of stimuli. Then the experimenter introduced the touch-screen search game, explaining that funny monsters were hiding under the big target circles, but not under the little circles. When a target circle was touched, a coloured square-shaped face covering approximately half the area of the target appeared and remained on display for the duration of the trial. This eliminated the requirement of remembering which targets had been previously found and therefore isolated differences in search from (possibly independent) memory differences. When a non-target circle (small or medium) was touched, nothing happened. The search continued until either 8 targets were found or the screen was touched 20 times. When the final target was touched or at the location of the 20th touch, a large face appeared for a few seconds and the search was terminated. After a demonstration by the experimenter, children did a practice run, during which they were verbally reinforced for touching targets and encouraged to look for more monsters. Children were then presented first with a baseline run with no distractors (to control for non-attentional differences in motor control and speed) and were then presented with the four experimental runs in randomised order across children. Each was preceded by a practice run.

### 1.4 Statistical analyses

Mean search time per hit (speed measure in seconds), mean distance between successive touches (path measure in centimetres), total number of errors (accuracy measure) and error types (touches on distractors, repetitions on previously found targets, and inaccurate touches on the screen) were calculated for each toddler. Errors were classified into three mutually exclusive categories: repetitive touches (e.g., due to children touching a previously found target); touches on distractors; and other erroneous touches (due to children touching the background near targets, rather than any of the targets or distractors). The current experiment differs from a standard search task in that hits and errors are intermixed in each run, whereas in traditional visual search task reaction times are calculated independently of false alarms and we therefore followed this strategy here. The speed and path measures were corrected for time and distance spent making errors, in order to obtain measures that would be less dependent on accuracy and error types. To correct time, we subtracted the time spent making errors and divided the remaining time by the total number of hits. To correct distance, we divided the total distance between successive touches (whether they were correct or not) by the total number of touches (excluding immediate repeats on targets, which did not accrue any distance).

Firstly, variables were tested for violations of the requirements for parametric statistics. When violations occurred appropriate corrections were used (e.g., Greenhouse-Geisser correction for violations of sphericity), but statistically significant effects were also tested non-parametrically. Dependent variables were entered in a mixed factorial ANOVA with distractor heterogeneity (homogeneous vs. heterogeneous) and target-distractor similarity (dissimilar vs. similar distractors, for heterogeneous displays these represent the minority of distractors of one type) as within-subject variables and group (FXS children or MA controls) as the between-subject variable. As search performance was evaluated using three different types of dependent measures (accuracy, speed and path), a Bonferroni correction was employed to reduce the likelihood of Type I errors, corrected *α* = .05/3 = .016. Variables measured during baseline runs (with no distractors) were also analyzed to establish whether any effects could be attributed specifically to the requirement to search amongst distractors.

## 2 Results

Table 1 represents the total number of targets found, total number of errors, different error types, search speed and path as a function of group, target-distractor similarity and distractor heterogeneity. The table also includes average performance on baseline runs and reports statistically significant main effects and interaction effects. In summary, children with FXS did not search amongst distractors more slowly or less systematically than MA controls (speed and path measure), but the overall pattern of errors produced distinguished the two groups. Children with FXS produced more repetitive errors, and their errors were more influenced by similar than by dissimilar distractors compared to MA controls. They also produced more inaccurate pointing errors. These trends were supported statistically as follows.

### 2.1 Total number of targets found and errors

There was a statistically significant main effect of target-distractor similarity on the total number of targets found. This was confirmed by a Friedman test, *χ*^2^ = 13.088, *p* = .004. Runs with distractors that were similar to targets resulted in overall fewer targets found compared to those with dissimilar distractors (on average, 6.9 hits and 7.4 hits per run, respectively). The effect of group on the number of targets found also displayed a trend towards significance, *F*(1, 24) = 4.532, *p* = .048 (driven by the fact that children with FXS tended to find fewer targets than MA controls), which did not however survive Bonferroni correction. Neither heterogeneity nor any of the interactions had any significant effect on accuracy, highest *F* value = 1.273, *p* = .267. Children with FXS and controls did not differ in the total number of accurate touches on baseline runs, *t*(12) = −1.552, *p* = .147.

In terms of overall errors, children with FXS produced more errors in total than MA controls (on average, 7.0 errors per run and 2.38, respectively). A significantly larger number of errors for children with FXS across all conditions were also confirmed through non-parametrics statistics (Mann–Whitney *U* Test, *p* < .01 for all comparisons). The groups also tended to differ in the number of errors on baseline runs, *t*(13.305, corrected for homogeneity violations) = 2.247, *p* = .042, with children with FXS committing more errors than MA controls (4.1 errors as opposed to .77 errors, respectively), a comparison that again did not survive Bonferroni corrections. However, we explored whether this trend towards baseline group differences in total number of errors could account for the large group differences in errors over the runs with distractors, and this was not the case. The main effect of Group on total errors remained statistically significant when baseline errors were used as a covariate, *F*(1, 23) = 13.122, *p* = .001. None of the other main effects or interactions reached statistical significance, highest *F* value = 1.834, *p* = .188.

Beyond the overall differences in total number of errors, the current analysis aimed to characterise potential group differences in error types, by subdividing them into repetitive errors, distractor errors and inaccurate touches. A more conservative criterion was therefore employed to control for the increased likelihood of Type I errors, corrected *α* = .05/3 = .016.

### 2.2 Repetitions on previously touched targets

**Fig. 2:**
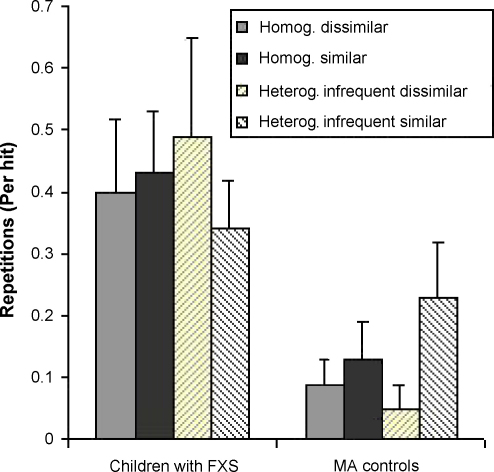
Average number of repetitive errors per hit by MA controls and children with FXS, as a function of target-distractor similarity and distractor heterogeneity (standard error of the mean is indicated in brackets).

Mean repetitions on previously found targets per run are represented in Fig. 2. Children with FXS produced more repeats on previously found targets than MA controls (on average, .491 repeats per hit and .129, respectively). These group differences were also tested using non-parametric statistics: children with FXS produced significantly more of these errors than controls in all four conditions, Mann–Whitney *U* Test, *p* < .01 for all comparisons. None of the other effects (similarity and homogeneity) and interactions reached significance, highest *F*(1, 24) = 3.243, *p* = .084. Children with FXS did not differ from MA controls in the number of repeats per hit on baseline runs, *t*(12.102, corrected for homogeneity violations) = 1.543, *p* = .148.

Repetitive touches on targets were further subdivided into immediate repeats, and returns to previous targets after other locations were visited, to explore differences in these types of errors previously reported for older children with FXS (Wilding et al., 2002) and these are reported in Table 2. Alpha was divided by the number of mutually exclusive types of repetitive errors, corrected *α* = .05/2 = .025. Table 2 presents mean number of different types of repetitive errors per hit across the different search displays. There was a statistically significant main effect of Group on immediate repetitions per hit, *F*(1, 24) = 6.654, *p* = .016, due to children with FXS producing a larger number of these errors per hit (.288, *SEM* = ±.05 on average) than MA controls (.112, *SEM* henceforth = ±.05 on average). There was also a marginally significant main effect of Group on returns on previously found targets, *F*(1, 24) = 6.000, *p* = .022, due to children with FXS producing a larger number of these errors per hit (.131 ±.03 on average) than MA controls (.017 ±.03 on average). None of the other main effects (similarity and homogeneity) or interactions reached statistical significance, highest *F* value = 3.537, *p* = .074. Groups did not differ in the number of immediate repeats or returns in baseline runs, *t*(24) = 1.462, *p* = .157 and *t*(12, corrected for homogeneity violations) = 1.648, *p* = .125, respectively.

### 2.3 Erroneous touches on distractors

**Fig. 3:**
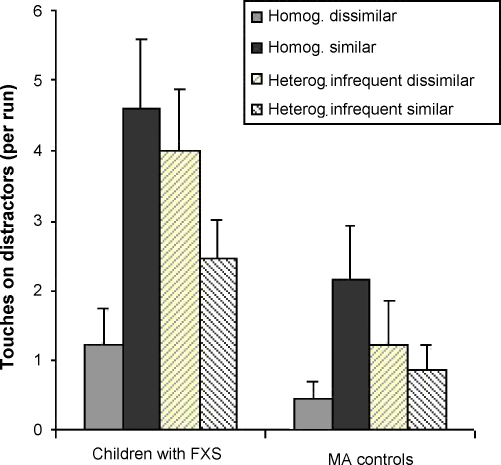
Average number of distractor touches per run by MA controls and children with FXS, as a function of target-distractor similarity and distractor heterogeneity (standard error of the mean is indicated in brackets).

Mean distractor touches per run are represented in Fig. 3. Children with FXS produced more touches on distractor circles than MA controls (on average, 1.5 and .59 errors of this type per run, respectively). There was also a main effect of target-distractor similarity, due to a greater number of distractor errors when distractors were similar (1.5 per run on average) than when they were dissimilar to targets (.63 per run on average). The main effect of similarity was confirmed using non-parametric statistics: there were statistically significant differences across conditions, Friedman test, *χ*^2^ = 13.830, *p* = .003, and fewer of these errors were committed in the condition with homogeneous dissimilar distractors than in the condition with homogeneous similar distractors, Wilcoxon Signed Ranks Test, *p* = .001. These differences seemed greater for children with FXS, Wilcoxon Signed Ranks Test, *p* = .008, than for MA controls, Wilcoxon Signed Ranks Test, *p* = .049 (with the latter not surviving correction for multiple comparisons). However, when tested using parametric statistics, none of the other effects and interactions reached significance, highest *F* value = 3.492, *p* = .074 for the interaction between similarity and group.

To explore further the type of distractor errors produced by all children, these were subdivided into touches on the smallest (most dissimilar) distractors, and those on the medium (similar) distractors across heterogeneous conditions. In these conditions, medium and small distractors were intermixed in different proportions, resulting in either similar or dissimilar distractors being more infrequent, and therefore more salient compared to the conditions in which they were frequent. Therefore, in addition to the absolute number of touches on each distractor type, we calculated the percentage of touches divided by the variable number of distractors of that type present in each display and we thank an anonymous Reviewer for the latter suggestion. Mean number of touches on similar and dissimilar distractors across all conditions and the percentage of these touches moderated by the overall number of each distractor type per display are presented in Table 3. Alpha was divided by the number of mutually exclusive types of distractor errors, corrected *α* = .025.

Let us first consider absolute mean number of distractor touches on similar and dissimilar distractors. In the heterogeneous condition with infrequent similar distractors (6 per display), all children incorrectly touched more frequently the similar compared to the dissimilar distractors, *F*(1, 24) = 29.170, *p* < .001, and children with FXS made more distractor touches overall, *F*(1, 24) = 5.906, *p* = .023. Critically, similarity of the distractors and Group interacted, *F*(1, 24) = 6.027, *p* = .022. This interaction effect was due to children with FXS incorrectly touching similar distractors more frequently (on average 2.15 times per run) than MA controls (on average, .77 times per run), *t*(24) = 2.496, *p* = .020 (Mann–Whitney *U* Test, *p* = .012), while the two groups did not differ in the number of times they touched dissimilar distractors, *t*(19.2, corrected for violations of homogeneity) = 1.50, *p* = .150 (.31 and .08 times per run, respectively, Mann–Whitney *U* Test, *p* = .289). In this heterogeneous condition, children with FXS also touched similar distractors more often than dissimilar distractors, *t*(12) = 4.951, *p* < .001 (Wilcoxon Signed Rank Test, *p* = .002), whereas MA controls only exhibited a trend in this direction, *t*(12) = 2.420, *p* = .032 (Wilcoxon Signed Rank Test, *p* = .041), which did not survive correction for multiple comparisons. When dissimilar distractors were infrequent, children with FXS generally touched distractors more than controls, *F*(1, 24) = 6.421, *p* = .018 (on average 2.0 and .62 of these errors for children with FXS and MA controls, respectively), but touches on similar and dissimilar distractors did not differ, *F*(1, 24) = 1.607, *p* = .205.

When one considers the number of distractors touched in the two heterogeneous displays in proportion to their number in each display (e.g., 6 distractors if infrequent, 18 distractors if frequent), a similar pattern of group differences emerges. When similar distractors were infrequent (6 per display), all children erroneously touched a higher proportion of these distractors (by touching 24.3% of distractors of these type, ±4.6) compared to dissimilar distractors (by touching 1.07% of dissimilar distractors in the display ±.43), *F*(1, 24) = 28.56, *p* < .001, and children with FXS touched a higher proportion of distractors overall (18.8% ± 3.48) compared to MA controls (6.62% ± 3.48), *F*(1, 24) = 6.153, *p* = .021. Additionally, Group and distractor type interacted significantly, *F*(1, 24) = 6.252, *p* = .020. This was due to children with FXS touching a significantly higher proportion of similar distractors (35.89% ± 6.54) compared to MA controls (12.82% ± 6.54), *t*(24) = 2.496, *p* = .020 (Mann–Whitney *U* Test, *p* = .012), and a significantly higher proportion of similar distractors compared to dissimilar ones, *t*(12) = 4.864, *p* < .001 (Wilcoxon Signed Rank Test, *p* = .002), This difference was statistically significant for MA controls, *t*(12) = 2.404, *p* = .033 (Wilcoxon Signed Ranks Test, *p* = .042), but did not survive correction by multiple comparisons. The two groups did not differ in the proportion of dissimilar distractors touched (1.71% ± .6 for children with FXS and .43% ± .6 for MA controls), *t*(19.200, corrected for heterogeneity of variance) = 1.5, *p* = .147 (Mann–Whitney *U* Test, *p* = .143). In heterogeneous displays in which dissimilar distractors were infrequent, children with FXS simply produced more distractor touches (20.09% ± 4.59) than MA controls (4.28% ± 4.59) overall, *F*(1, 24) = 5.936, .023. Finally, children with FXS were more affected than controls by the relative proportion of distractors of various sizes, producing more touches on similar distractors when they were infrequent than when they were frequent, *t*(12) = 2.851, *p* = .015 (Wilcoxon Signed Ranks Test, *p* = .007). This difference was not statistically significant for controls, *t*(12) = 1.298, *p* = .219.

### 2.4 Inaccurate touches

**Fig. 4:**
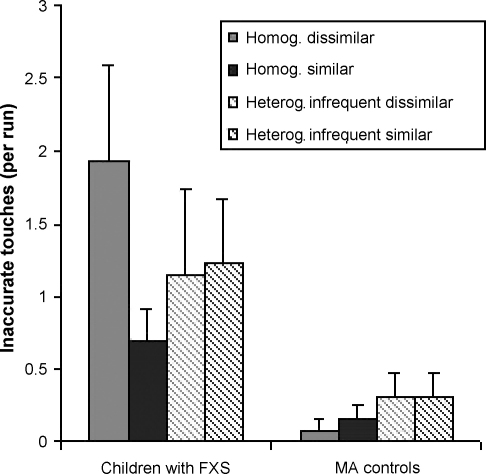
Average number of inaccurate touches per run by MA controls and children with FXS, as a function of target-distractor similarity and distractor heterogeneity (standard error of the mean is indicated in brackets).

Fig. 4 illustrates the average number of inaccurate touches per run by group and search condition. There was a main effect of Group on the number of inaccurate touches in the experimental trials, due to children with FXS producing a larger number of these errors per run (1.250 ± .24 on average) than MA controls (.212 ± .24 on average). This was also confirmed through non-parametric statistics, showing statistically significant differences across groups when homogeneous similar distractors were present, Wilcoxon Signed Ranks Test, *p* = .001. The effect of homogeneity and the other interaction effects did not reach statistical significance, highest *F* value = 2.678, *p* = .115. Groups did not differ in terms of inaccurate touches in baseline runs, *t*(13.488) = 1.793, *p* = .095.

### 2.5 Analyses of search speed and path

Target-distractor similarity, distractor heterogeneity and group membership did not affect search speed differentially in the two groups. None of the interaction effects reached statistical significance, highest *F* value = 2.919, *p* = .1. Furthermore, children with FXS and MA controls did not differ in speed to find targets on baseline runs, *t*(24) = 1.498, *p* = .147. Similarity, heterogeneity and group did not affect search path significantly, highest *F* value = 2.177, *p* = .152, but there was a trend towards statistically significant interaction between similarity and group, *F*(1, 24) = 4.730, *p* = .040, which did not however survive Bonferroni corrections. However, children with FXS produced significantly larger search paths than MA controls on baseline runs, *t*(24) = 4.863, *p* < .001 (Mann–Whitney *U* Test, *p* < .001). We therefore explored whether this difference in search path in runs that did not contain distractors could influence group differences in distractor runs by employing baseline distance between successive touches as a covariate in the earlier analysis of variance. None of the main effects or interaction effects in this additional analysis reached statistical significance, lowest *p* = .146.

## 3 Discussion

For the first time, the current experiment investigated in detail the vulnerability to multiple factors affecting target and distractor salience by young children with FXS and younger mental-age matched typically developing controls. Children with FXS did not differ from controls in their overall search speed or strategy when searching amongst distractors, but the larger number of errors and specific error types distinguished them from typically developing children very clearly. They made more errors than controls by repeatedly touching targets that had already been found and their false alarm rates to non-targets were more influenced by the similarity of targets and distractors and by the relative salience of distractors than was the case for typically developing controls. Children with FXS also produced a larger number of inaccurate touches. We review these error types in turn, and evaluate how they may relate to underlying atypical computations in fragile X syndrome.

The pattern of repetitive errors by young children with FXS provides further support for their striking deficits in inhibitory control (Cornish et al., 2001; Cornish, Scerif, & Karmiloff-Smith, in press; Munir et al., 2000; Scerif et al., 2004, 2005; Wilding et al., 2002). These errors can be accounted for by the difficulty in suppressing a previously correct but now inappropriate response. They are entirely consistent with an understanding of how fragile X syndrome affects the development of neurocomputational properties relevant to executive control. First, recurrent connections in frontoparietal cortices rely on excitatory glutamatergic inputs (Elston, 2002, 2003). Thus, activity in areas proposed to be crucial for inhibition and goal maintenance may depend on some of the structural and functional properties compromised in FXS. Indeed, imaging data suggest atypical functioning of distributed frontal, parietal and striatal circuits in FXS (Menon et al., 2004; Tamm et al., 2002). Secondly, modulation of extrinsic and intrinsic neurotransmitters key to executive control functions depends on the fine structure of the dendritic spines on which these inputs converge (Gao & Goldman-Rakic, 2003), making them potentially more vulnerable to the abnormalities characteristic of FXS. Thirdly, precursors of extrinsic neurotransmitters themselves seem to be regulated by FMRP, suggesting their abnormal neuromodulation (Gruss & Braun, 2004; Zhang et al., 2005). Indeed, deficits that have thus far been most often associated with extrinsic neurotransmitter dysfunctions have also been reported in FXS (e.g., differences in baseline eye-blink rate, Roberts, Symons, Johnson, Hatton, & Boccia, 2005).

Importantly, the current findings do not suggest that the neurocognitive profile of young children with fragile X syndrome is characterised by selective abnormalities in isolation, as could be the case in normal adults who suffered discrete brain lesions. Indeed, with the current experiment, for the first time, we measured both repetitive behaviours and concurrent effects of the similarity of targets and distractors, the heterogeneity of distractors and their relative ratio in each search display. We found multiple atypical effects of these factors for children with FXS. Children with FXS were more affected by distractors that were similar to targets than control children were, as previously demonstrated by Scerif et al. (2004). These difficulties might appear surprising given the lack of distractor errors in older children with FXS required to discriminate targets and distractors on the basis of simple orientation and colour combinations (Wilding et al., 2002). Greater errors detected here may well relate to difficulties in representing the relative size difference between targets and distractors. Why should this process be affected by fragile X syndrome? Computations of relative size/magnitude depend on parietal circuits (Pinel, Piazza, Le Bihan, & Dehaene, 2004), which resemble prefrontal cortices in their dendritic spine complexity (Elston, 2003). Furthermore, parietal cortices receive afferents from magnocellular thalamic input, and recent studies have revealed differentially greater expression of FMRP in magnocellular, rather than parvocellular neurones of the lateral geniculate nucleus of the thalamus (Kogan, Boutet, et al., 2004). These may in turn impact the development of the functions of parietal cortices to which they project, although longitudinal relationships across processes have not been studied.

In addition, for the first time here we showed that children with FXS were more likely to erroneously touch distractors that were similar to targets when they were infrequent, compared to controls. Infrequent distractors receive greater attentional weight in models of item salience because computations of salience depend on the difference between an element in the search display and every other element in the display (Cave & Wolfe, 1990) and between that element and the neighbouring elements (Wolfe, 1994), making infrequent elements more salient. This difference has been employed to account for the strong effects of the ratio between different types of distractors on search efficiency (Shen et al., 2000). The fact that children with FXS committed greater numbers of erroneous distractor touches to infrequent distractors that were similar to targets compared to controls suggests that, in addition to their difficulties in processing target-distractor similarity, they processed the increased salience of similar distractors differently and/or were less capable of ignoring it by allocating greater attention to target stimuli.

Although all children were affected by the similarity of targets and distractors, they did not benefit from homogeneity of distractors in terms of either search speed or path as adults do (Humphreys et al., 1989). This may be because distractor grouping benefits tend to be smaller even in adults when search items are presented in irregular configurations, rather than placed in a regular array (Humphreys et al., 1989). Alternatively, it may be that displays defined by the size of local items are processed differently in young children compared to adults, as has been suggested for young infants (Sireteanu, Encke, & Bachert, 2005). We thank an anonymous reviewer for pointing out that, however, children erroneously touched distractors of each type less frequently in homogeneous than in heterogeneous displays (if one takes into account their relative proportions), suggesting an improvement in the accuracy of their search with homogeneity. Children with FXS did not differ from controls in how vulnerable they were to overall changes in distractor heterogeneity, as predicted by the fact that perceptual grouping by older children with the syndrome is in line with their overall developmental level (Cornish et al., 1999). Therefore, limited effects of overall heterogeneity are again consistent with other characteristics of the syndrome.

Finally, our touch-screen based search task also revealed motor inaccuracies in pointing in young children with fragile X syndrome. Fine motor control difficulties have been reported by clinicians and parents (Hagerman & Hagerman, 2002), and they have been assessed longitudinally using the Vineland Adaptive Behavior Scales or through video-based observation (e.g., Baranek et al., 2005). The current findings support these clinical and parental reports. Published studies have not yet studied systematically whether there are direct implications of children's difficulties in fine and gross motor control and their differences in perceptual processing of stimuli under attentional demands (e.g., difficulties in estimating distances and negotiating movements in everyday complex environments). Additionally, it remains to be investigated whether basic differences in functional vision (e.g., first and second order motion thresholds, Kogan, Bertone, et al., 2004; Kogan, Boutet, et al., 2004) or subtle and controversial ocular abnormalities affecting crowded acuity (e.g., cf. Hatton, Buckley, Lachiewicz, & Roberts, 1998; Maino, Wesson, Schlange, Cibis, & Maino, 1991) could affect children's ability to evaluate and correctly select stimuli in their environment, and in turn impact on their motor control skills. Longitudinal studies encompassing perceptual, attentional and motor development are needed to establish relationships across these developing functions.

Having reviewed individual characteristics of search performance by children with FXS, it remains an important challenge to also account for their co-occurrence. Firstly, why do these multiple deficits (repetitive errors, erroneous distractor touches, inaccurate pointing) co-occur in FXS, and is this unique to this condition? We propose that answers depend on investigating thoroughly the changes in neurocomputational constraints imposed by FMR1 silencing, as has been argued more generally for studies linking genetics and cognition (Fisher, 2006). FMR1 silencing imposes gross changes on dendritic spine morphology and physiology, as well as on neurotransmitter regulation. Critically, as these processes are all dynamically involved in activity- and age-dependent changes, they need to be placed within a developmental context, both at a cellular level (Lim, Booker, & Fallon, 2005) and at the cognitive level (Scerif et al., 2005; Scerif & Karmiloff-Smith, 2005). Secondly, considering the association of deficits reported here raises a further issue. Although the particular combination of error types reported here characterises FXS, none of the individual error types in isolation need to be unique to the condition. For example, toddlers with another genetic disorder, Williams syndrome, were affected to an even greater degree by target-distractor similarity in size when searching for targets amongst distractors, although they did not produce equally large numbers of repetitive errors (Scerif et al., 2004). Indeed, both cross-syndrome similarities and differences suggest that there may be multiple atypical pathways to deficits in search performance specifically, and attentional control more generally (Cornish et al., in press).

In conclusion, the complex profile of search performance in a genetically well-characterised monogenic disorder such as fragile X syndrome should not be surprising, if one integrates the neurocognitive profile of FXS with the molecular and systems neuroscience of FMRP. The co-occurrence of these deficits, some more apparent than others, highlights the need to focus on understanding the cascade of complex developmental effects associated with the silencing of single genes (Fisher, 2006), from the genetic level of description to relatively uneven cognitive profiles, as is consistent with a dynamic view of neurocognitive development (Karmiloff-Smith, 1998). We argue that this requires a focus on understanding how low-level changes in neurocomputations may be differentially more relevant to certain cognitive functions compared to others, rather than on the pursuit of selective mappings between genes and high-level cognitive processes themselves. More generally, the findings stress the role of genetic disorders in an integrated and multidisciplinary framework aimed at unveiling the complex links between genomics, cellular/systems neuroscience and the development of high-level cognition.

## Figures and Tables

**Table 1:** Average number of errors, erroneous touches on distractors and repetitive errors on previously found targets as a function of group, target-distractor similarity and distractor heterogeneity (standard error of the mean is indicated in brackets)

Dependent variables	Distractor types		Baseline	Homog. dissimilar	Homog. similar	Heterog. (infrequent dissimilar)	Heterog. (infrequent similar)	Statistically significant effects	*F* ratio, *p* value
Search accuracy									
Accurate responses (per run)	FXS		7.23 (.49)	6.85 (.54)	6.0 (.55)	6.38 (.56)	6.84 (.54)	Similarity	*F*(1, 24) = 9.973, *p* = .004
	MA		8.0 (.00)	7.92 (.08)	7.46 (.39)	7.61 (.31)	7.77 (.23)		
Total errors (per run)	FXS		4.15 (.17)	6.0 (1.15)	7.54 (1.01)	7.46 (1.16)	7.3 (1.11)	Group	*F*(1, 24) = 26.593, *p* < .001
	MA		.77 (.34)	1.16 (.59)	3.0 (1.0)	2.0 (.68)	2.92 (.77)		
	Repetitions (per hit)	FXS	.69 (.41)	.40 (.12)	.43 (.10)	.49 (.16)	.34 (.08)	Group	*F*(1, 24) = 7.788, *p* = .01
		MA	.06 (.03)	.09 (.04)	.13 (.06)	.05 (.04)	.23 (.09)		
	Touches on distractors (per run)	FXS	NA	1.23 (.51)	4.62 (.97)	4.0 (.89)	2.46 (.55)	Group	*F*(1, 24) = 15.885, *p* = .001
		MA	NA	.46 (.24)	2.15 (.77)	1.23 (.62)	.85 (.37)	Similarity	*F*(1, 24) = 21.125, *p* < .001
	Inaccurate touches (per run)	FXS	1.46 (.67)	1.92 (.67)	.69 (.23)	1.15 (.59)	1.23 (.44)	Group	*F*(1, 24) = 9.763, *p* = .005
		MA	.23 (.17)	.08 (.08)	.15 (.10)	.31 (.17)	.31 (.17)		

Search speed (s)									
	FXS		1.33 (.13)	1.84 (.51)	1.54 (.18)	1.74 (.24)	1.81 (.24)		
	MA		1.63 (.15)	1.55 (.18)	2.17 (.24)	1.63 (.21)	1.58 (.19)		

Search path (cm)									
	FXS		7.76 (.45)	6.67 (.58)	5.18 (.39)	5.53 (.38)	5.58 (.45)	Group (baseline condition only)	*t*(24) = 4.863, *p* < .001
	MA		4.99 (.35)	4.89 (.32)	5.32 (.41)	5.57 (.29)	5.01 (.26)		

**Table 2:** Average number of immediate repeats on previously found targets per hit and returns to hits as a function of group, target-distractor similarity and distractor heterogeneity (standard error of the mean is indicated in brackets)

Type of repetition error	Distractor type	Baseline	Homog. dissimilar	Heterog. (infrequent dissimilar)	Heterog. (infrequent similar)	Homog. similar
Immediate repeats (per hit)	FXS	.60 (.37)	.29 (.07)	.36 (.11)	.19 (.06)	.31 (.08)
	MA	.06 (.03)	.09 (.04)	.05 (.04)	.19 (.09)	.13 (.06)

Returns (per hit)	FXS	.09 (.06)	.11 (.08)	.14 (.07)	.15 (.06)	.12 (.05)
	MA	.00 (.00)	.00 (.00)	.01 (.01)	.05 (.02)	.01 (.01)

**Table 3:** (a) Average absolute number of distractor touches per run on medium (similar) and small (dissimilar) distractors, respectively and (b) proportion of targets of that type touched when search displays contained both types of distractor: heterogeneous displays with infrequent similar distractors or heterogeneous with infrequent dissimilar distractors

Type of distractors	Distractor condition	Homog. dissimilar	Homog. similar	Heterog. (infrequent similar)	Heterog. (infrequent dissimilar)
Similar distractors					
Similar distractors per display		0	24	6	18
Touches on similar distractors (per run)	FXS	–	4.62 (.98)	2.15 (.45)	2.39 (.68)
	MA	–	2.15 (.77)	.77 (.32)	1.08 (.63)
Touches on similar distractors (per run, %)	FXS	–	19.23 (4.1)	35.89 (7.5)	13.25 (3.8)
	MA	–	12.82 (5.4)	5.98 (3.5)	8.98 (3.2)

Dissimilar distractors					
Dissimilar distractors per display		24	0	18	6
Touches on dissimilar distractors (per run)	FXS	1.23 (.31)	–	.30 (.13)	1.62 (.76)
	MA	.46 (.24)	–	.08 (.08)	.15 (.10)
Touches on dissimilar distractors (per run, %)	FXS	5.13 (2.1)	–	1.71 (.74)	26.92 (12.7)
	MA	1.92 (1.0)	–	.43 (.43)	2.56 (1.7)

The mean number of distractor touches for the homogeneous conditions are also provided for reference (standard error of the mean is indicated in brackets).
